# Changing Trends in Maxillofacial Trauma: A Single-Center Retrospective Study in India

**DOI:** 10.1055/s-0045-1802556

**Published:** 2025-02-03

**Authors:** Kapil S. Agrawal, Vivek Gupta, Mudunuri Ravi Teja, Raghav Shrotriya, Vinita A. Puri

**Affiliations:** 1 Department of Plastic Surgery, Seth GS Medical College and KEM Hospital, Mumbai, Maharashtra, India

**Keywords:** maxillofacial trauma, facial injury trend, facial trauma epidemiology, craniomaxillofacial surgery

## Abstract

**Background:**

Maxillofacial trauma is quite commonly encountered either in isolation or in association with polytrauma. The present observational study aims to analyze the changing trends of maxillofacial injuries and mull over some probable reasons for the same.

**Materials and Methods:**

This is an observational retrospective study done at a tertiary care center in Mumbai, India, for a period of 12 years (2008–2019) after getting clearance from the institutional ethics committee. All patients who were admitted in the plastic surgery unit with maxillofacial trauma were included in the study and data were collected from case record sheets in the archives of the department. The data obtained were tabulated and analyzed.

**Results:**

A total 1,046 patients were included in the study. The most common age group involved was 21 to 30 years (50.19%). Males outnumbered females in terms of hospital admissions (92.7 vs. 7.3%). Road traffic accidents (RTA) were found to be the major etiological factor (72.27%). Out of 756 RTA victims, 533 (70.5%) were due to two-wheeler accidents. In the present study, the zygomaticomaxillary complex (middle third) was most commonly fractured (40.73%) followed by the mandible (38.91%). Panfacial fractures (i.e., those involving at least two facial thirds) comprised 10.71% of all the cases.

**Conclusion:**

The incidence of maxillofacial trauma is showing an increasing trend in the recent past. The majority of the patients are victims of RTAs and two-wheeler accidents are increasingly responsible for such injuries. The classical injury patterns and the fracture patterns that were described in the past are not routinely observed now. The injury and fracture pattern itself has become more complex. We observed a statistically significant raise in midface fractures in our study.

## Introduction

The face is the most exposed part of the body and is very prone to trauma, causing injury of skeletal components, dentition, or soft tissues of the face. 1 Maxillofacial trauma is quite commonly encountered in a trauma setup either in isolation or in association with polytrauma. Most of the causes of the maxillofacial fracture can be grouped into either road traffic accidents (RTAs), falls, or assaults. 2 According to the World Health Organization (WHO) statistics, nearly 25% of all injury fatalities are due to RTA, and 90% of fatalities occur in low- and middle-income countries. 3 The incidence of RTAs has been steadily falling in developed countries, but are rising in low- and middle-income countries. 4 Maxillofacial injuries have a great impact and put a burden on the patient in terms of quality of life and lost man-days. Over the past two decades, it has been felt that the character of the injuries and the injured has been undergoing a gradual change over time in India. Despite being common in clinical practice, little is known and researched about the changing trends with chronology.

The primary objective of this study was to measure the changing trends in etiology of trauma and the changing injury pattern over time, and the incidence of fractures with respect to two-wheelers and four-wheelers and with respect to use of protective gears. Another objective is to determine the relationship between the use of head protective gear while driving a two-wheeler and the incidence of maxillofacial injuries.

## Materials and Methods

This is an observational retrospective study conducted in the Department of Plastic and Reconstructive Surgery, Seth GS Medical College and KEM Hospital, Parel, Mumbai, India. The study protocol was reviewed by the institutional ethics committee and ethical clearance was obtained to investigate further (Ethics No. EC/OA-147/2019). All patients of maxillofacial trauma who were admitted in the plastic surgery unit were included in the study over a span of 12 years (January 2008–December 2019). Data were obtained from the in-patient records (indoor and OT registers, discharge papers, procedure notes, clinical photographs, imaging records) of patients admitted with maxillofacial injuries during the aforementioned time period obtained from the medical records department and the archives of our department.

The patients for whom the data were incomplete and those who did not consent to regular follow-up and clinical photography were excluded. In cases of RTAs, the following parameters were evaluated: type of vehicle (two- or four-wheeler, heavy vehicle) being driven by the patient or whether the patient was a pedestrian hit by an automobile, type of passenger (driver or pillion rider), usage of protective gear like seat belts, helmets, and history of driving under the influence of alcohol or psychoactive substance. The presence of other associated injuries and their nature in cases of RTA with polytrauma were also evaluated. In the cases with facial fractures, the pattern of fractures, anatomical site, treatment type (observation vs. intervention), type of fracture reduction (open vs. closed approaches), and outcome measures like uneventful postoperative period or any postoperative complications and their management were analyzed. The yearly total number of maxillofacial injuries treated by us during the aforementioned time span was calculated and plotted graphically. Every year, two-wheeler and non-two-wheeler injuries were also plotted on graphs apart from the total number of yearly injuries.

The data collected included demographics such as age, gender of the patient, ethology, and pattern of skeletal involvement. The data were stored and analyzed using Microsoft Excel. The results were presented as simple frequencies and simple statistics. A *p* -value of less than 0.05 was considered significant.

## Results

**Fig. 1:**
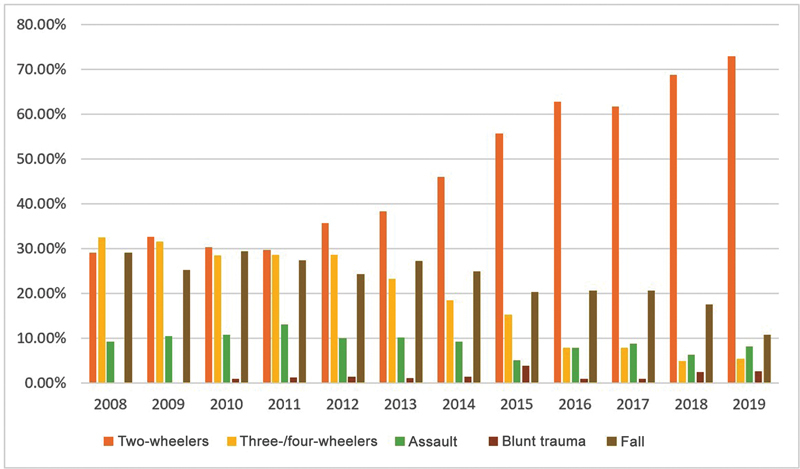
Trend of injury patterns over the years. A *p-* value of <0.001 was considered statistically significant.

The total number of patients included in the study was 1,046. The most common age group was 21 to 30 years ( *n* = 525; 50.19%). Males outnumbered females in terms of hospital admissions (92.7 vs. 7.3%). In all, 252 patients (24.09%) had a history of being under the influence of alcohol at the time of injury. RTAs were found to be the major etiological factor (756; 72.27%). Out of these, 533 patients (70.5%, *n* = 756) were victims of two-wheeler accidents, 203 patients (19.41%) were involved in four-wheeler accidents, and 20 (1.91%) were pedestrians hit by automobiles. Out of 533 patients of two-wheeler accidents, 463 patients (86.87%) were driving the motor vehicle and out of the 203 patients of four wheeler accidents, 105 (51.72%) were driving the vehicle and 76 (37.44%) were front-seaters adjacent to the driver. In our study, only 1.94% (9 out of 463) victims of two-wheeler injuries were wearing a helmet. It was interesting to see that there is a statistically significant increase ( *p* < 0.001) in two-wheeler accidents as a proportion of the total etiology over the years ( Fig. 1 ), while the other etiological factors remain relatively on a decreasing trend. We observed that 44% of two-wheeler drivers were under the influence of alcohol at the time of accident as opposed to 39% in three-/four-wheeler drivers.

In terms of the anatomical location, the zygomaticomaxillary complex (ZMC) was the most common fracture (40.73%), followed by the mandible (38.91%) in the present study. Panfacial fractures (i.e., those involving at least two facial thirds) comprised 10.71% of all the cases ( Table 1 ). Classical Lefort fracture patterns were observed in less than 5% (46 patients). There was a history of concomitant injuries like head injuries, limb and thoracoabdominal injuries along with facial fractures in 62.52% of the cases ( Table 2 ). The most common associated injury was head injury in 29.64% of cases. About 15.7% of cases had multiple associated injuries along with facial fracture and approximately 2.01% of cases sustained a spinal injury.

**Table 1:** Fracture distribution

Facial bone	No. of fractures	Percentage
Mandible (lower one-third)	407	38.91
Midface (middle one-third)	426	40.73
Frontal (upper one-third)	101	9.65
Panfacial	112	10.71

**Table 2:** Associated injuries

Associated injuries	No. of patients (1,046)	Percentage
Limb injury	77	7.36
Head injury	310	29.64
Thoracic	41	3.92
Abdominal	40	3.82
Spine injury	21	2.01
Multiple injury	165	15.51
Nil	392	37.48

Most of our patients required open reduction and internal fixation (ORIF) for their facial fractures. Only 4.78% of the cases were managed conservatively with soft diet, rest, and antiedema measures. Unless contraindicated, fiberoptic nasal intubation with a north pole tube is the preferred route of intubation in our department. In total, 83.8% of cases were intubated through the nasal route, followed by the orotracheal route in 14.4% of cases. In only 17 patients (0.017%), tracheostomy, which was done for other indications, was used to secure the airway during surgery ( Table 3 ). In 153 patients (14.8%), intermaxillary fixation was required postoperatively, of which 121 patients suffered mandible fractures (predominantly condylar and subcondylar fractures), 19 suffered panfacial injuries, and 13 had maxilla fractures.

**Table 3:** Type of intubation

Intubation	No. of patients (996)	Percentage
Nasal	835	83.8
Orotracheal • Retromolar • Submental	144 33 23	14.4
Tracheostomy	17	0.017
Total	996	100

**Fig. 2:**
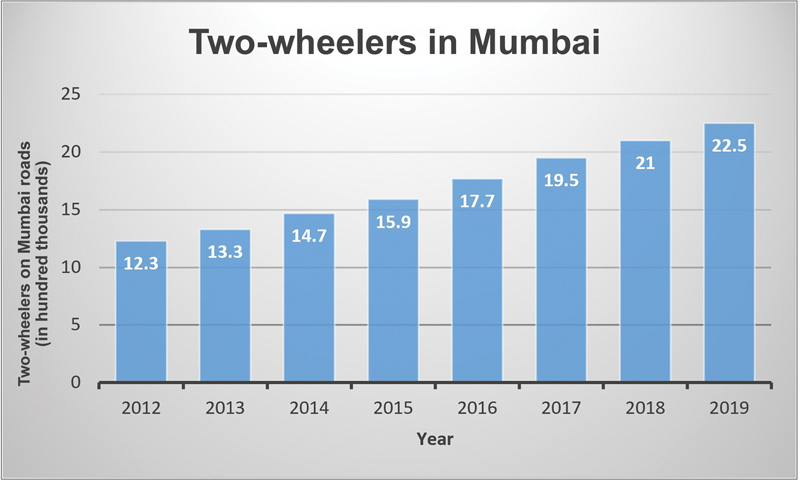
Two-wheelers on Mumbai roads represented in hundred thousands. (This image is provided courtesy of RTO Office, Mumbai).

**Fig. 3:**
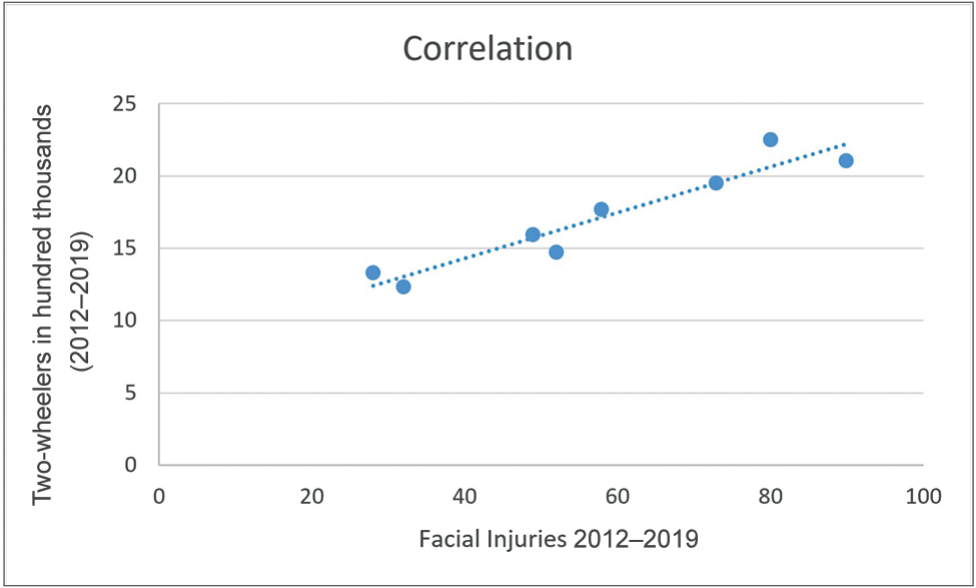
Scatter plot showing a positive correlation between increasing number of two-wheelers and increasing facial fractures.

**Fig. 4:**
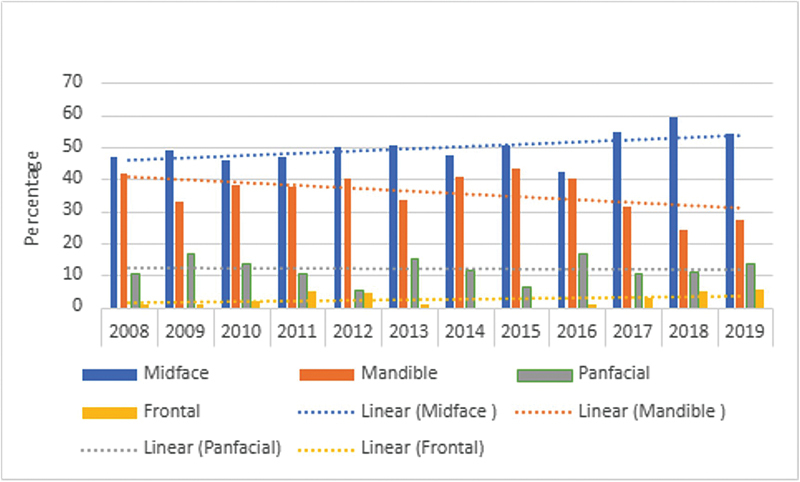
Trend of fracture patterns over the years.

The yearly total number of maxillofacial injuries treated during the study period was calculated and plotted graphically, and the following observations were made. The incidence of maxillofacial trauma is showing an increasing trend in the recent past. Most of the patients were victims of RTAs and that too two-wheeler accidents, and the incidence of two-wheeler accidents is taking a rising curve as compared with the incidence of maxillofacial trauma due to non-two-wheeler accidents and it was statistically significant ( Fig. 1 ). As per the statistics obtained from Regional Transport Office (RTO) in Mumbai, there is an increasing trend in the footfall of two-wheelers on Mumbai roads ( Fig. 2 ). In our study, we found a positive correlation between increasing two-wheelers and increasing two-wheeler injuries ( Fig. 3 ). In our study, the classical injury patterns and the fracture patterns that were described in the past were not routinely observed now. The injury and fracture pattern itself has become more complex. When we tried to observe trends in facial fracture patterns over the years, we noticed that the incidence of midface fractures increased from 46.6% in 2008 to 54.2% in 2019, whereas the incidence of mandible fractures decreased from 41.6% in 2008 to 27.2% in 2019 ( Fig. 4 ). On applying linear regression analysis on the overall trends of facial fractures, we found the following:

- **Frontal:** Slight increase (0.21% per year); not statistically significant ( *p* = 0.2326).
- **Mandible:** Decrease (–1.44% per year); borderline significance ( *p* = 0.0538).
- **Midface:** Increase (1.29% per year); statistically significant ( *p* = 0.0498).
- **Panfacial:** No significant trend ( *p* = 0.8849).

**Fig. 5:**
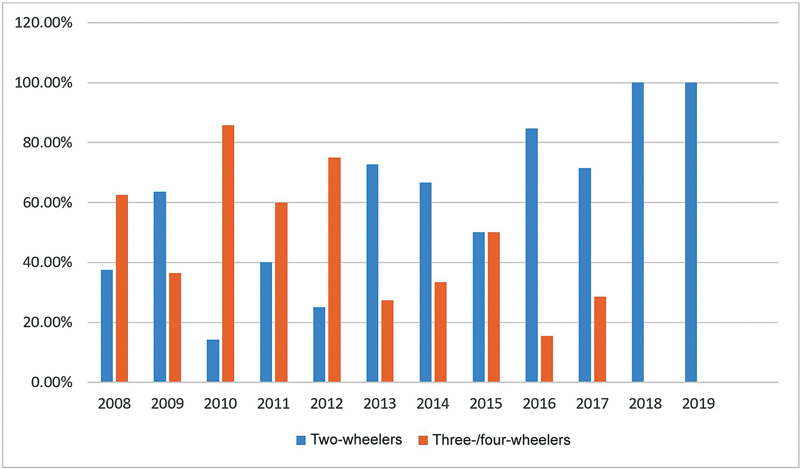
Annual trends in panfacial trauma in road traffic accidents. A *p* -value of 0.017 was considered statistically significant.

These results suggest that midface fractures have shown a statistically significant increase over the years, while other classifications do not show significant trends. We found a similar trend in RTA cases for midface and mandible fractures, but we have observed that there is a statistically significant increase in panfacial fractures with two-wheeler accidents as compared with four-wheeler accidents over the years ( *p* = –0.017; Fig. 5 ). Although the trend of fracture pattern in non-RTA cases (fall, assaults, blunt injuries, etc.), which comprised approximately 34.4% of our sample size, was variable and statistically not significant, we found the majority of fractures occurred in the mandible (48.3%), followed by the midface (38%), which was contrary to our observation in the overall sample size.

**Fig. 6:**
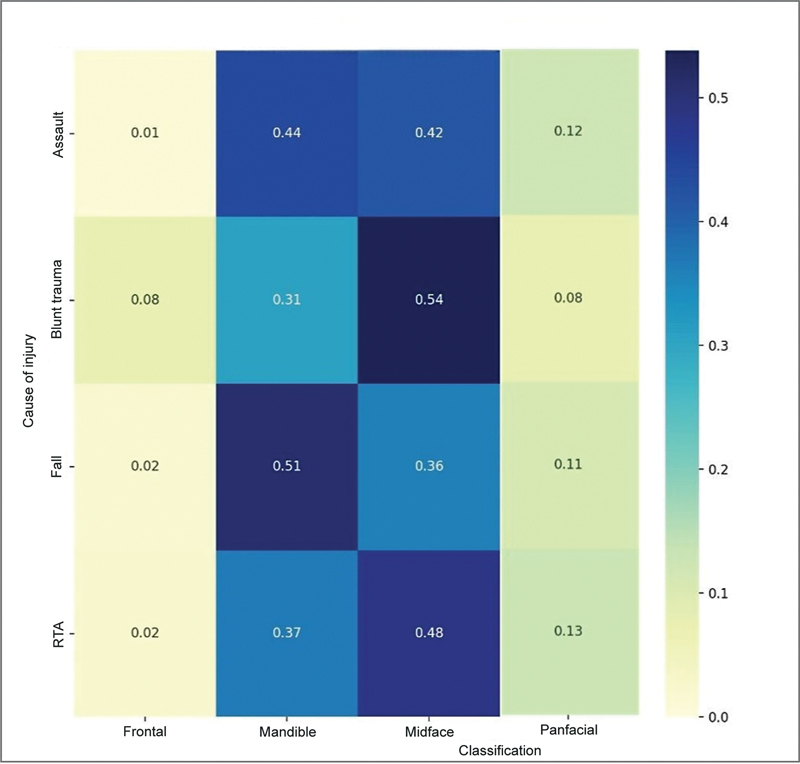
Correlation heat map: cause of injury versus classification of fracture. The vertical axis represents the causes of injury. The horizontal axis shows the classification of fractures. The color intensity and the number in each cell represent the correlation between a specific cause of injury and a type of fracture. *Darker blue colors* indicate stronger positive correlations, while *lighter colors* suggest weaker correlations. RTA, road traffic accident.

Using modern statistical tools, we plotted a correlation heat map for the cause of injury versus classification of the fractures sustained ( Fig. 6 ). Some of our key observations are the following:

- RTAs:
  – Strongly correlated with midface fractures (0.54).
  – Moderately correlated with mandible fractures (0.32).
  – This suggests that RTAs are a major cause of facial injuries, particularly affecting the midface region.
- Falls:
  – Highest correlation with mandible fractures (0.46).
  – Also showed a notable correlation with midface fractures (0.36).
- Assaults:
  – Strongly correlated with mandible fractures (0.58).
  – This indicates that in cases of assault, the mandible is the most frequently affected area.
- Blunt trauma/sports injuries:
  – Highest correlation with midface fractures (0.54).
  – This suggests that sports-related injuries tend to affect the midface region more than other areas.

Although the above observations were made out of the correlation heat map, we could not get any statistically significant correlation or predictability.

## Discussion

In our study, the most commonly injured age group was 21 to 30 years. Males outnumbered females. These were comparable to other studies in the existing literature. 5 In a meta-analysis from Saudi Arabia, 10 to 21 years was the most common age group getting injured. 6

RTAs emerged as the main cause of maxillofacial trauma. Two-wheeler injuries are the most common and outnumber all other non-two-wheeler injuries put together. The reasons for higher frequency of RTA in developing countries could be lack of adequate knowledge about road safety measures, callous attitude of the road users, which include pedestrians, cyclists, motorists, and vehicle passengers, toward complying with road safety measures, unsuitable road conditions, speed limit violation, use of old and refurbished vehicles with improper safety features, poor street lighting, low driving standards, more two-wheelers than the road can accommodate, defective layout of crossroads and speed breakers, narrow roads and heavy trafﬁc, fast- and slow-moving vehicles in the same lane, overloaded vehicles, neglecting use of protective gears, and violation of highway code. 7 In our study, only 1.94% (9 out of 463) victims of two-wheeler injuries were wearing a helmet and none had a chin protector included in it ( Table 1 ). This points toward a strong protective effect of helmet for a two-wheeler and seatbelt for a four-wheeler, as has been found in some previous studies. 8 In terms of noncompliance with usage of protective gear, especially helmets, there is a lack of uniform regional rules pertaining to their use, reluctance to use due to the myth of causing hair loss, 9 and fear of theft, all of which could be a few contributory factors. According to a study by Bhoi et al, motorcyclists are five times more likely to have a severe head injury if they do not wear helmets and three times more likely to die than those who do. 8 Traveling in a four-wheeler equipped with airbags and fastening seat belts while traveling could decrease the incidence and severity of RTA-related injuries, 7 thus making two-wheelers inherently more unsafe as compared with four-wheelers.

In terms of the anatomical location, midface fractures (41%cases) and mandible fractures (39% of cases) are the predominant type of fractures in our study ( Table 1 ). There is a statistically significant increase in the trend of midface fractures over the years in our study. This finding is slightly different from some other reports. 7 In a recent study conducted Chandra et al in Delhi-NCR, isolated mandibular fractures accounted for 48.6% cases, followed by midface fractures (27.6%). 5 However, Singaram et al, 10 Subhashraj et al, 11 and Septa et al 12 observed that midfacial fractures were more common than mandible fractures, especially the zygomatic bone and arches, which found the mandible as the most commonly fractured bone. The high incidence of ZMC fractures may be related to frontal trauma in cases of RTA and may be commensurate with the increasing incidence of two-wheeler-related trauma.

This study highlights few trends observed in the pattern of incidence of maxillofacial injury patients presenting to our department.

The number of patients presenting to our casualty ward with maxillofacial injuries has steadily increased over the years and has doubled over the 12 years of our study duration (60–120 patients).

It was observed in this study that maxillofacial trauma patients are presenting at a much younger age than before. The average age in 2008 was 38.4 years as compared with 23.6 years in 2019. This observation coincides with the increasing proportion of two-wheeler-related injuries as found in our study.

Over the years, the patients involved in two-wheeler accidents have contributed an increasing proportion to the maxillofacial fractures requiring hospital admission and surgical treatment. 13

Over the years, the fractures have become more complicated. Although ZMC fractures and mandibular fractures are the most common fractures over the years, there has been a statistically significant increase in the ZMC fractures ( Fig. 4 ) and decrease in mandible fractures. Among the two-wheelers, there is a disproportionate and rising trend in panfacial fractures ( Fig. 5 ). This may be related to the changing etiology as high-speed two-wheeler trauma has become the dominant etiological factor in these cases. In a 2003 study, Kim et al showed that the incidence of panfacial fractures was 6.5% as compared with our study, which showed it was 10.7%. 14 This may be related to the fact that their data were from 1997 to 2001.

Trauma care in India is evolving rapidly in leaps and bounds owing to drastic reduction in morbidity and improved survival in patients with polytrauma. But measures designed to prevent RTAs are still in their infancy. This observed pattern of increasing incidence of maxillofacial injuries due to RTAs year after year indicates that stringent measures from the concerned authorities to decrease the incidence of RTAs and improve road safety are the need of the hour. Few of those could be to enforce strict laws like mandatory use of seatbelts and total headguard (that suit climatic conditions) rather than the conventional helmets. If we consider RTAs as an epidemic of modern times, then road safety measures should be the vaccine. Public awareness campaigns regarding this epidemic will go a long way toward sensitizing the people about this problem. Public should be educated that compliance to road safety measures is for their own safety rather than to avoid being fined. Better policing, strict implementation of trafﬁc laws, and observation of trafﬁc rules would help considerably reduce the incidence of RTAs. 15 Education about road safety using innovative methods, particularly in the most vulnerable age group (15–45 years), will also help in prevention.

This study has some limitations like information bias as there could be some missing information when using existing records. The valuable information regarding the occupation and educational background of the patient, the victim's adherence to road safety measures apart from use of protective gear, condition of the vehicles involved in the RTA, and the condition of the road where RTA had occurred were not available. There could be the possibility of selection bias because the patients included in the study had maxillofacial injuries due to RTAs and those who did not have any maxillofacial facial injuries due to RTA were not compared. Patients who were treated on an outpatient basis were not included in the study. Prospective studies in this regard with complete information on the factors pertaining to victim, vehicle, and road preferably with uniform and standardized proforma are recommended to statistically validate the data.

## Conclusion

This retrospective study demonstrates an increasing trend in maxillofacial trauma over a 12-year period, largely driven by a significant rise in two-wheeler accidents. While the overall incidence of maxillofacial trauma has increased, this trend aligns with the growing presence of two-wheelers on the roads, contributing to higher-velocity collisions and more complex injury patterns. However, it is important to recognize that other etiological factors, including four-wheeler accidents and falls, continue to contribute to facial traumas.

Our study reveals a significant paradigm shift in injury patterns, characterized by a pronounced increase in midface fractures relative to mandibular fractures. Furthermore, the proportion of panfacial fractures is higher in two-wheeler accidents compared with other modes of transport. These observations highlight the evolving nature of maxillofacial trauma, underscoring the imperative need for a comprehensive, multipronged approach to effectively address this dynamic and pressing public health concern.

To mitigate the growing burden of maxillofacial trauma, it is imperative to implement strengthened road safety measures. This includes more stricter enforcement of traffic regulations, improvements in road infrastructure, and comprehensive public awareness campaigns. Promoting the consistent use of protective gear, such as helmets, is particularly critical in reducing the severity of injuries and enhancing overall safety.
